# Spatio-temporal variability of processes across Antarctic ice-bed–ocean interfaces

**DOI:** 10.1038/s41467-018-04583-0

**Published:** 2018-06-18

**Authors:** Florence Colleoni, Laura De Santis, Christine S. Siddoway, Andrea Bergamasco, Nicholas R. Golledge, Gerrit Lohmann, Sandra Passchier, Martin J. Siegert

**Affiliations:** 1 Fondazione Centro Euro-Mediterraneo sui Cambiamenti Climatici, 40129 Bologna, Italy; 2 Istituto Nazionale di Oceanografia Sperimentale, 34010 Sgonico, Italy; 3 Department of Geology, Colorado College, Colorado Springs, Colorado 80903 USA; 4 Centro Nazionale delle Ricerche - Istituto di Scienze Marine, 30122 Venice, Italy; 5 Antarctic Research Centre, Victoria University of Wellington, Wellington, 6140 New Zealand; 6 GNS Science, Avalon, Lower Hutt, 5010 New Zealand; 7 Alfred Wegener Institute, Helmholtz Centre for Polar and Marine Research, 27570 Bremerhaven, Germany; 8 University of Bremen, 28359 Bremen, Germany; 9 Department of Earth and Environmental Studies, Center for Environmental and Life Sciences, Montclair State University, Montclair, NY 07043 USA; 10 Grantham Institute and Department of Earth Science and Engineering, Imperial College of London, London, SW7 2AZ UK

## Abstract

Understanding how the Antarctic ice sheet will respond to global warming relies on knowledge of how it has behaved in the past. The use of numerical models, the only means to quantitatively predict the future, is hindered by limitations to topographic data both now and in the past, and in knowledge of how subsurface oceanic, glaciological and hydrological processes interact. Incorporating the variety and interplay of such processes, operating at multiple spatio-temporal scales, is critical to modeling the Antarctic’s system evolution and requires direct observations in challenging locations. As these processes do not observe disciplinary boundaries neither should our future research.

## Abstract (web summary)

Understudied in the Antarctic system are the subsurface interfaces between ice-sheet, ocean and geological substrate. Here, the authors review our understanding of these components and propose new avenues of holistic dynamic modeling to achieve a unified understanding of past, present and future polar climate.

## Introduction

The Antarctic Ice Sheet (AIS) is at risk of reaching a tipping point (critical threshold for irreversible large-scale changes caused by small perturbations) under the COP21 + 2 °C atmospheric warming limit^1^ by around 2030–2050^2^. Once this threshold is reached, global sea-level rise of several, possibly tens, of meters on timescales of centuries to millenia becomes inevitable^3,4^. Currently, ice-sheet models disagree on the origin and magnitude of the main processes driving AIS retreat and on the proportion of its contribution to projected sea-level rise by 2100 and beyond^4–6^, as well as in the past^7,8^.

Over the last decade, ocean heat supply to the continental shelves bordering the Southern Ocean has been shown to be the main cause of thinning and retreat observed on several floating Antarctic ice shelves^9^, with atmospheric temperature rise playing a relatively minor role. It has been observed and modeled that in areas where the bed slopes toward the continental interior, ice shelf thinning can lead to a Marine Ice-Sheet Instability (MISI)^10,11^. Both numerical simulations and observations show that if the buttressing support of the floating ice shelves is removed from the grounded ice, ice-sheet flow to the ocean may be enhanced^12^, leading to accelerated and substantial mass loss in a few years to a few decades^13^. Direct measurements of physical processes and their feedbacks leading to MISI are difficult to aquire over the spatio-temporal scales of glaciological changes, however. Alternatively, past evidence, testifying to repeated rapid retreats of both the East and West components of the AIS (EAIS and WAIS) over the past 5 million years^14^, provides a valuable basis for validation of the physics of numerical climate and ice-sheet models, allowing calibration between the past and future in our assessments of ice sheet evolution. As a consequence, the Intergovernmental Panel on Climate Change commissioned a special “Oceans and Cryosphere” report to urgently identify the knowledge gaps concerning the ice–ocean interactions.

**Fig. 1:**
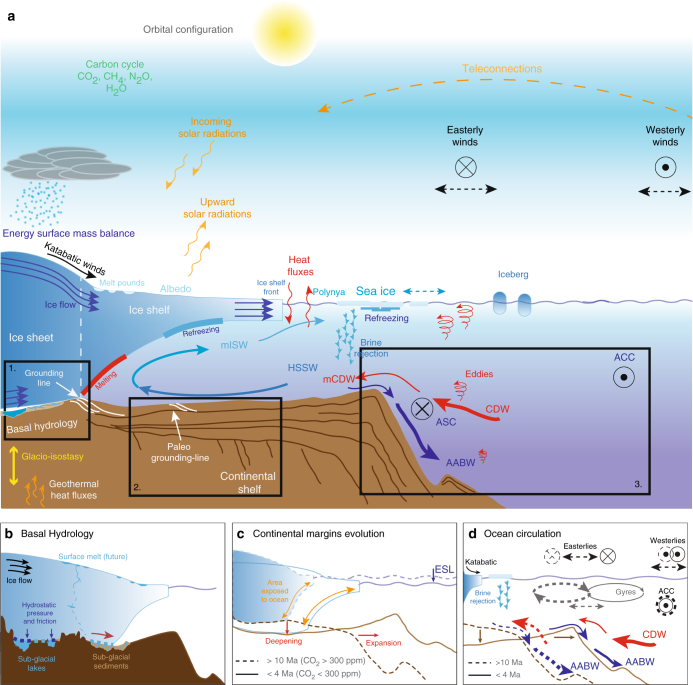
Conceptual and simplified view of the Antarctic polar system. **a** The state-of-the-art and the knowledge gaps about subglacial hydrology (1), continental shelf evolution (2), and ocean circulation (3) are discussed in the present review. **b** Macro-scale (e.g., mountains and lakes) and micro-scale (presence of deformable sediments) subglacial bed roughness influences the relationship between ice sheet and hydrological processes. Basal meltwater can saturate sediments, which helps to accelerating the ice flow and impacts on grounding line dynamics. **c** Geophysical records suggest that the modern seafloor reached its landward dipping morphology and extent by the late Miocene-early Pliocene ( ≈ 5 Ma), as a result of intense subglacial erosion. **d** Numerical simulations^89^ suggest that a smaller Weddell Sea continental shelf, as during the mid Miocene ( ≈ 15 Ma) compared to modern, induces a poleward shift of regional oceanic circulation, and enhances heat transport (CDW/AABW) across the continental shelf edge. The depth and shape of the continental shelf edge and slope determine where the intrusions of CDW occur. The sub-cavity ocean processes and the glacio-isostatic adjustment will be discussed in upcoming reviews by Smith et al. (in preparation) and Whitehouse et al. (in preparation). AABW, Antarctic Bottom Water; ACC, Antarctic Circumpolar Current; ASC, Antarctic Slope Current; CDW, Circumpolar Deep Water; GL, Grounding line; HSSW, High Salinity Shelf Water; mCDW, modified CDW; mISW, modified Ice-Shelf Water. Note that the oceanic processes represented in 2D view for the purpose of the illustration might not occur at the same locations on the continental shelf

Numerical studies have shown that kilometer to sub-kilometer spatial resolution is needed to simulate grounding zone migration of ice shelves and outlet glaciers^15^, and to calculate the intermittent and highly-localized incursions of oceanic warm waters across the continental shelf break, partly caused by short-term mesoscale eddy formation (Fig. 1) and/or tides^16^. The lack of a detailed representation of bed morphology beneath grounded and floating ice, and of seabed morphology across continental shelves, resolved well enough to properly simulate the essential processes and interactions, hampers our ability to describe past major changes^17^, constrain tipping points and assess rates of previous and future sea-level changes^7^ (Fig. 1).

**Fig. 2:**
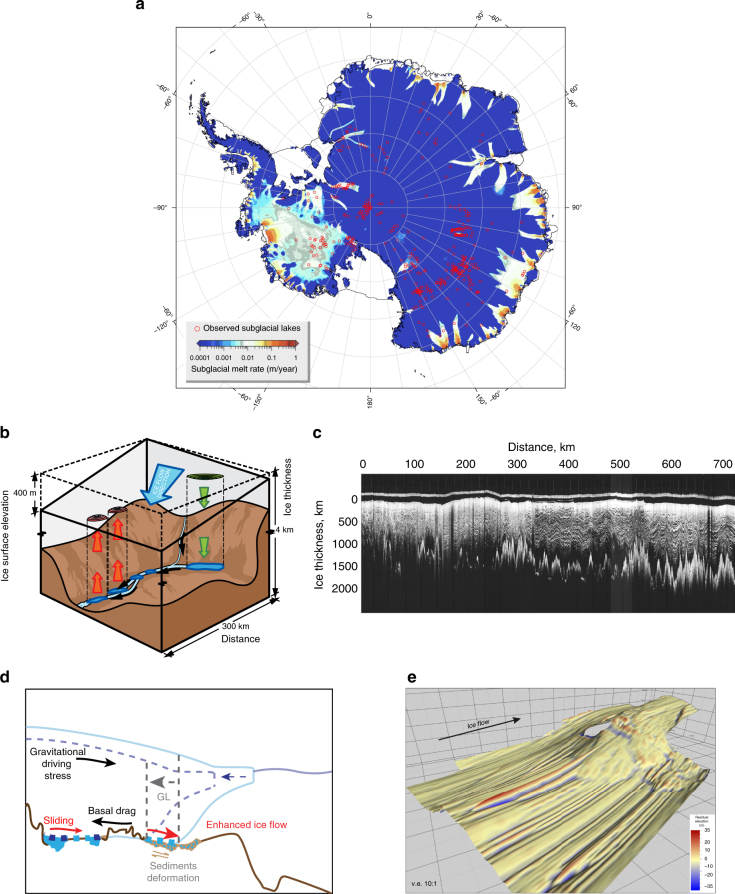
Key elements of suglacial hydrology. **a** Current subglacial lakes locations^115^ (red circles) superimposed on the rate of present-day basal meltwater production (ma^−1^) simulated with the Parallel Ice Sheet Model at 5 km resolution (standard run, unpublished). **b** An illustration of rapid drainage and hydraulic connection of Antarctic subglacial lakes, inferred from satellite altimetric measurements of the ice-sheet surface over the Adventure Subglacial Trench, East Antarctica, between 1996 and 2003^27^. **c** Airborne radio-echo sounding transect across the Gamburtsev Subglacial Mountains in central East Antarctica. The transect was acquired by the Scott Polar Research Institute as part of a collaboration between the UK, USA, and Denmark in the late 1970s. The transect itself reveals mountains over 1 km in relief buried beneath the thick ice of the polar plateau. **d** Relationship between ice-sheet driving stresses, basal drag, basal sliding, and subglacial hydrology. Sliding induces a reduction in basal drag and accelerates ice flow. **e** Sedimentary landforms indicative of fast-flowing grounded ice beneath the Rutford Ice Stream, as imaged from ice-penetrating radar^43^. Vertical exaggeration is 10 × . Sedimentary bodies such as these significantly influence the dynamics of the Antarctic ice sheet by changing the topography of the bed and/or by enhancing sliding at the ice bed interface. Figure 2b has been adapted with permission from Springer Nature; Nature volume 440, pages 1033–1036, Duncan J. Wingham, Martin J. Siegert, Andrew Shepherd, Alan S. Muir (20 April 2006) doi:10.1038/nature04660. Figure 2ewas reproduced from Figure 4 of King, E. C., Pritchard, H. D., and Smith, A. M.: Subglacial landforms beneath Rutford Ice Stream, Antarctica: detailed bed topography from ice-penetrating radar, Earth Syst. Sci. Data, 8, 151-158, https://doi.org/10.5194/essd-8-151-2016, 2016; https://creativecommons.org/licenses/by/3.0/

Processes operating in cavities beneath ice shelves impact large-scale and long-term retreat, or partial collapse, of the AIS (to be discussed in an upcoming review by Smith et al. in preparation). The resulting glacio-isostatic adjustment induces feedbacks that enhance or dampen ice retreat on various spatial and temporal scales (to be discussed in an upcoming review by Whitehouse et al., in preparation) (Fig. 1). In this review, we examine: (1) the importance of subglacial bed morphology and hydrological conditions, as a control on ice-sheet flow (Fig 1a); (2) the way continental shelf morphology influences AIS dynamics and ice-sheet sensitivity to ocean forcing (Fig. 1b); and (3) how ocean exchanges, and in particular the oceanic heat supply to AIS margins (Fig. 1c), operates at a variety of spatial and temporal scales. These processes take place within the subsurface environment of both the ice sheet and the ocean, and at the physical interfaces between them, which necessitates cross-disciplinary research to observe, measure, and understand them. These three physiographic realms (subglacial, continental shelf, and ocean) are understudied, yet critical to forming knowledge of AIS evolution from the deep past to the future (Fig. 1).

## Subglacial water and basal ice-flow processes

Ice-sheet flow is controlled by processes acting at its bed. Although ice flow can occur slowly by the deformation of ice, it is sliding over an ice-rock interface or deformation of weak water-saturated basal sediments that mainly dictates the flux of ice to the ocean. Sliding of ice sheets is constrained by bed roughness at a variety of scales from the macro (i.e. bed topography, Fig. 2d) to the micro-scale^18^). Basal water, where present, may lubricate the base of an ice sheet or a glacier, causing ice flow acceleration^19^ and enhancing erosion of the bed^18^. Depending on bed morphology and conditions, subglacial water is now understood to exist in three ways (Fig. 2b): (1) subglacial lakes, which are large stores of water existing in isolated or connected bed depressions; (2) organized channels that route subglacial water, which may be cut down into the bed or carved upwards into the overlying ice; and (3) subglacial aquifers, in which water infiltrates through dilatant sediments or flows in thin films at the ice bed interface.

Thanks to radio-echo sounding (RES) campaigns, more than 400 subglacial lakes, scattered across the continent, have been identified to date^20,21^ (Fig. 2a). Their bright, smooth specular radio-wave reflections are distinct from those over ice-rock (Fig. 2c) or ice-sediment interfaces^22^. Although the heat for the meltwater that feeds subglacial lakes is not known well, geothermal heat and that developed from basal friction, as well as pressure melting point decrease from hydrostatic pressure, are the main factors (Fig. 2d). How water flows beneath the ice sheet is critical to the dynamics of ice above. The dominant control on water flow is the basal hydro-potential gradient, which is a function of ice surface slope, with bed slopes being an order of magnitude less significant^23^. As a consequence, water melted beneath the center of an ice sheet is routed to its margins^24,25,^ where drainage may occur^26,27^ over a period of weeks and months. Hence, there is an association between the fast-flowing ice streams (and sliding) and the availability of water at their beds^28,29^. On longer timescales, groundwater could accumulate in subglacial aquifers during glaciations^30^ and be released during interglacials, when the ice sheet thins and retreats, inducing further ice flow changes.

Basal motion is a key component of the total velocity solution for a modeled ice mass, and depends on both macro- and micro-scale roughness and basal resistance (dragging stresses) of the bed^18^ (Fig. 2d). Some ice-sheet models parameterize the subglacial boundary in a way that allows basal drag to evolve in space and time as a function of meltwater availability and sediment cohesion^31^ (Fig. 2d). This formulation is based on a Coulomb-type flow law^32^, which allows substrate deformation to be characterized by either a linear response to applied stress, a purely plastic response, or by exhibiting some intermediate behavior between these end members. Sliding then depends on effective pressure and allows basal drag to reduce smoothly to zero, instead of a step change in basal drag at the floating ice transition^33^. Although model comparison studies show that this transition improves grounding-line tracking, the artificially imposed smoothing along the grounding line might not be valid everywhere.

Despite localized high-resolution bed observations for some areas, the current low spatial resolution of subglacial topography from BEDMAP2^34^ precludes detailed knowledge of basal water pathways over most of Antarctica and impedes precise simulations of basal hydrology within ice-sheet models. As micro-scale roughness is largely unknown, ice-sheet models use satellite observations of surface velocities in fast-flowing areas to “invert” for total basal friction, enabling ice stream dynamics to be simulated^35^. Continent-wide models have achieved success with simulations using this and related techniques^5,36^. Inverting ice velocities does not provide explicit information on the basal hydrology nor on past or future changes in basal conditions. Consequently, when climate conditions depart too much from present-day state and lead to substantial expansion or retreat of the AIS away from its present-day margins, this technique fails. Hence, there is an urgent need to refine the parameters for basal hydrologic processes in ice-sheet models. A hydrological scheme bridging the gap between relatively small-scale geophysically observed phenomena and their representation in continental-scale ice flow models has been implemented and tested within the Parallel Ice Sheet Model^37^. This mass-conservative scheme includes a subglacial deformable sediment layer combined with a distributed system of linked, water-filled cavities that open as a consequence of sliding and close due to the creep of ice over a range of spatial and timescales. To validate the physics of this scheme, present-day simulations should predict locations of at least the larger (> 10 km long) subglacial lakes.

Some conceptual experiments have produced realistic simulations of ice flow over subglacial lakes using simplified geometry ^38^ or for a limited area domain^39^. At a continent scale, the best results will be achieved once high-resolution bed topography, geothermal heat flux distribution^40–42^, and the location and thickness of subglacial sediments are known well. More direct observations of these are needed to account for hydrological processes and to improve ice-sheet models. A great utility of RES observations is in the identification and location of basal water, and mechanisms by which water may flow. RES methods are incapable of sounding through water, however, and there is a lack of information beneath suglacial water surfaces. This shortfall can be addressed by other methods such as sonar, gravity inversion, and seismic surveying that may reveal the thickness and extent of soft water-saturated basal sediments^43,44^ (Fig. 2e).

The need for high-resolution subglacial bed topography and seabed bathymetry is demonstrated by recent work that reveals a relict hydrological network dating back to the last glaciation and evidence for grounding line retreat across the Ross Sea shallow continental shelf^45^. Cross-cutting relationships between fluvial channels and grounding zone wedges also show that hydrological networks evolve through time^46,47^, as a consequence of glacial dynamics changes and of erosion of the subglacial bed itself. Hence, there is much knowledge of modern hydrological processes to be gained from the study of paleo ice-sheet beds. Simulation of robust basal hydrology that incorporates distributed subglacial lakes and channel networks, localized by regional- to local-scale bed morphology, calls for flexible models that replicate transient and/or evolving conditions arising from a change in shape of the bed due to erosion or deposition, and from variations in effective pressure resulting from the presence of basal water. To attain a continental-scale picture of basal hydrological processes, and to verify current hydrological observations, spatial gaps that exist in the current subglacial and seabed morphology must be filled. Robust and accurate past subglacial bed and seabed morphology reconstructions (e.g., the RAISED Consortium^48^) are needed to simulate realistic past basal hydrology^49,^ and thus ice flow, in order to constrain past AIS dynamics.

## Continental shelf evolution at the ice bed–ocean interfaces

**Fig. 3:**
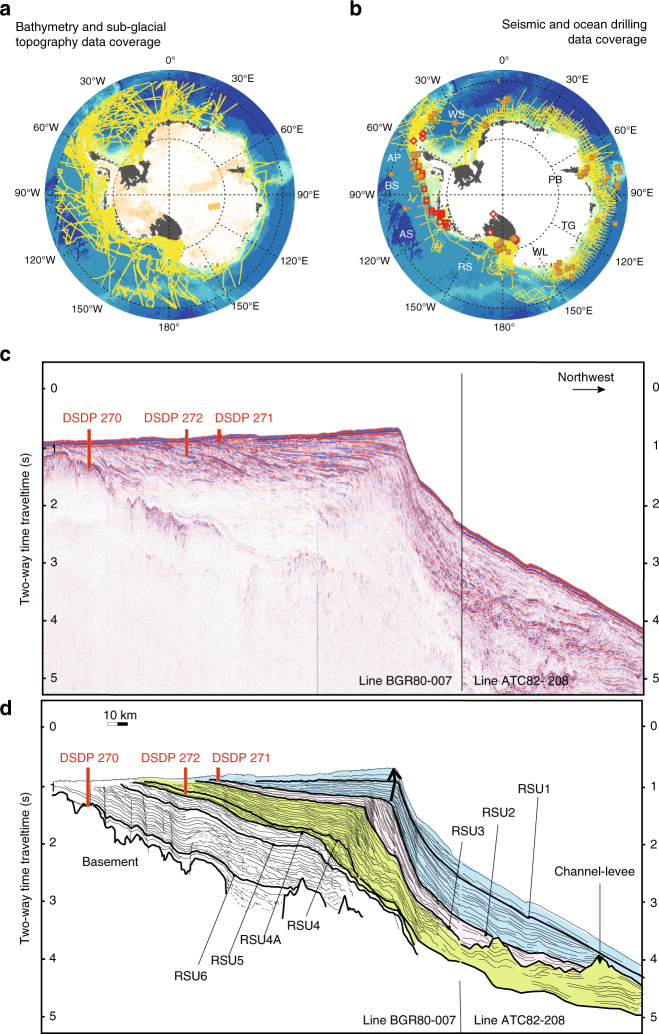
Present and past knowledge of continental margins morphology. **a** Bathymetric (yellow) and subglacial topography (orange) data coverage used to reconstruct the BEDMAP2 subglacial topographic dataset^34^ including the bathymetry around the margins from the IBCSO dataset^56^ (here, multibeam coverage only). **b** Seismic data coverage (yellow, Antarctic Seismic Data Library System, https://sdls.ogs.trieste.it) from Antarctic Peninsula (AP), Amundsen Sea (AS), Bellingshausen Sea (BS), Ross Sea (RS), Weddell Sea (WS), Wilkes Land (WL), Prydz Bay (PB) and Sabrina Coast-Totten Glacier (TG) continental margins. Existing (yellow squares) or short-term scheduled (red) Ocean Drilling Program/International Ocean Discrovery Program drilling sites (https://iodp.tamu.edu/scienceops/maps.html) used to constrain and restore past continental margins morphology. White or gray areas indicate little or no data coverage. **c** Multichannel seismic profiles BRG80-007 and ATC82-208 in the Ross Sea with existing deep drill site locations (projected) from Deep Sea Drilling Program Leg 28^116^. **d** Line drawing^52^ showing the horizons from the Oligocene-early Miocene Ross Sea Unconformity RSU6 to the Pleistocene RSU1 across the eastern Ross Sea. Progradation of the continental shelf margin occurred from RSU4A to RSU3 (early-late Miocene, 19-10 Ma^51^^,^^52^), along with channel-levee complexes formation in the deeper areas (green shade). During the next phase between RSU3 and RSU2 (late Miocene-early Pliocene, 10–4 Ma^51^^,^^52^), trough-mouth fans formed on the continental slope and sedimentation rates gradually decreased in the continental shelf and rise (pink shade). The change from a prograding to aggrading continental shelf edge (indicated by the arrows) is observed after RSU2 from early to mid-Pliocene (4–3.7 Ma), when the shelf profile became landward deepening

Reconstructions of past subglacial and seabed morphology of the continental margins of Antarctica represent a challenge for the paleo-polar community. Multibeam bathymetry^45,50^ and seismic stratigraphy^51–54^ reveal evidence for widespread past erosional and depositional modifications to the seabed over the last 34 Ma. These data show that sedimentary units deposited in the Pliocene (5–3 Ma) were eroded during late-Pleistocene glaciations in many places. Despite this evidence, paleo ice-sheet simulations of key climate intervals commonly use modern subglacial bed topography and seabed bathymetry from BEDMAP^55^, BEDMAP2^34^, and IBCSO^56^ as boundary conditions if no other paleo-reconstructions are available^57^. However, inherent inaccuracies arising from heterogeneous spatial data coverage (Fig. 3a) introduce uncertainties into numerical simulations that strive to identify past and future AIS tipping points^58^. Paleo-ice-sheet simulations will be improved when robust reconstructed paleo-bed topography is provided to the modeling community.

For areas having dense seismic and deep drilling data coverage (Fig. 3a), sediment backstripping is a powerful technique for bathymetric reconstructions. Sediment properties and thickness provide constraints for decompacting accumulated sediments (see Box 1 below). By successively removing sediment layers, and by isostatically rebounding the underlying surface and through consideration of the tectonics, the depth and extent of continental shelves at times of maximum glacial advance may be inferred. To reconstruct paleo-depths and continental shelf morphology between two maximum glacial advances, spatial sediment erosion has to be quantified. Sediment isopach maps^59–61^ combined with seismic stratigraphy and drilling sites are used to trace back the eroded sediments to their source position^62^. Uncertainty in those reconstructions is large and depends on the spatial data coverage. For some key regions in the Ross Sea^52^ and Weddell Sea^63^, detailed topographic and seabed reconstructions have been achieved for times of past climate significance. In the case of the Ross Sea, backstripping and depth calculations for Miocene units robustly show a change in sedimentary deposition from shallow and seaward dipping to overdeepened and landward dipping continental shelves^52^ (Fig. 3b).

The correct interpretation of backstripping reconstructions relies on the knowledge of past sedimentological and climatic history of the continental shelf environments. Marine seismic sections from the Antarctic Peninsula, Amundsen Sea, Bellingshausen Sea, Ross Sea, Weddell Sea, Wilkes Land margins, Sabrina Coast-Totten Glacier, and Prydz Bay continental margins (Fig. 3a), correlated to marine cores and geological records since ≈ 34 Ma, show an alternation of ice proximal and ice distal, glacial and interglacial marine facies on the continental shelf with large channel-levee complexes present in the deeper areas^51,61,64–67^. This indicates that the AIS was highly dynamic under temperate climate conditions of the Oligocene-early Miocene (Fig. 3b green shade; Box 1, time t1).

By the mid–late Miocene (≈ 14 Ma), global climate cooling led to the expansion of grounded ice and the cold-based area underneath the AIS^65,68^, which favored AIS stability, with fluctuation of AIS margins over only a narrow zone on the outer continental shelf. The formation of trough-mouth fans at the continental shelf edge and a decreased sedimentation rate on the rise^69^ both indicate extensive subglacial sediment erosion and glacial marine deposition on the outer shelf and upper slope. In several locations, progradational wedges imaged seismically show that an expansion of the continental shelves occurred before the mid-Pliocene (Fig. 3b, pink shade). An aggradation of the shelf followed with deepening of the landward slope (Fig. 3b, blue shade; Box 1, time t2). Concomitant deposition of sediment drifts indicates that the Antarctic Slope Current (ASC, Fig. 1) actively contributed to the maintenance of channel levees that were shaped mainly by local turbidity currents in the deeper areas of the pan-Antarctic continental margin^70,71^.

The dynamic geological history of the AIS margins clearly demonstrates how past continental shelf morphologies differed from the modern one. However, a continent-wide paleotopographic reconstruction, partly achieved using backstripping, exists for just one important climate interval, so far, at ≈ 33.8–33.5 Ma^57,62,66^. The time of the reconstruction is for the presumed onset of continental glaciation at the Eocene-Oligocene Transition (EOT). Other climate transitions of specific interest for paleogeographic reconstructions include the mid-Miocene (≈ 14 Ma), mid-Pliocene (≈ 3.3 Ma), and some of the late Pleistocene super interglacials (e.g., MIS 31 ≈ 1.1 Ma, MIS 11 ≈424 ka, MIS 5 ≈128 ka) or glacials (e.g., Last Glacial Maximum, LGM ≈ 21 ka). These times, marked in some cases by a substantial retreat of the AIS, may yield insights that allow the cryosphere community to identify upper and lower bounds for past WAIS and EAIS tipping points that may be surpassed in the future. This objective creates an impetus to develop Antarctic bathymetry/bed topography reconstructions for these selected intervals.

To achieve pan-Antarctic paleobathymetric reconstructions of these key periods, and reduce uncertainties in basal morphology, precise age and environmental information (e.g., past ice proximal versus distal lithological deposits, paleo-water depth, and eustatic sea level), paleo-landscape, and identification of pan-Antarctic seismic horizons of regional extent, will be required. Some regional maps of key Cenozoic horizons and sequences have recently been published^54,59–61,63,72^, however, the paucity of stratigraphic constraints hinders the comprehensive pan-Antarctic correlation of known horizons and pan-Antarctic bathymetric reconstructions. The drilling expeditions by the International Ocean Discovery Program scheduled in 2018–2020 will greatly expand knowledge of the WAIS margin (Fig. 3a).

Additional geophysical and drilling campaigns will be necessary to fill gaps in circum-Antarctic coverage and provide pan-Antarctic past boundary conditions to ice-sheet models. Recent numerical studies^17,57,73^ highlight the large spread in simulated ice volumes and extents produced by the uncertainties, or the lack of definition, of past bed morphologies. For example, simulations of AIS dynamics across the EOT using a maximum, mostly emergent, topography lead to larger ice volume and extent than when using a minimum, more subdued topography. Finally, the variations in bed over time likely had consequences for the AIS response to atmospheric and ocean forcing. When tested in ice-sheet simulations, AIS sensitivity to changes in ocean temperature increases along with a gradual deepening of the continental shelves. This is because the area of ice shelves that is exposed to ocean heat increases with a deepening of the bathymetry^74^.

### Box 1  Backstripping  technique

Paleobathymetry of a continental margin can be calculated using backstripping techniques^75^ that restore tectonic subsidence, decompact sediment, and remove water load for a given time. The backstripping procedure consists of stripping off stratigraphic units in sequence from the youngest to the oldest, calculating the decompaction of remaining underlying sedimentary units, and isostatic rebound after removal of upper sedimentary units, at each step. The depth at the time of sediment burial (*t*_pd_, *t*_1_, or *t*_0_) is restored for each horizon (red and yellow), deposited during the post-rift history (*t*_0_). The paleo-depth calculation takes into account the sedimentary units age (*t*_pd_, *t*_1_, or *t*_0_) and thickness (*S*), water ($\rho _{\mathrm{w}}$), sediment ($\rho _{\mathrm{s}}$), and mantle ($\rho _{\mathrm{m}}$) density, paleo-water depth (*W*_d_), and eustatic sea-level changes relative to present day (ΔSL). Isostatic rebound, caused by removal of sediment units and of water load, is usually calculated with either local (no rigidity and effective elastic thickness *T*_e_ = 0) or flexural (*T*_e_ > 0) isostasy.

Sediment decompaction is calculated considering the exponential decay of porosity (*ϕ*) with depth, for each sediment type. Glacial environments undergo an additional local sediment overcompaction due to the advance of grounding ice over the sediments, accounted for using the physical properties of sediments measured at drill sites (Fig. 3a) and extrapolated laterally as representative of marine seismic facies^76^. In those calculations, lithospheric rigidity and *T*_e_ are often set to fixed values for an entire area, although those variables may exhibit strong lateral variations (e.g., *T*_e_ = 30–60 km in the Ross Sea^77^). In the case of rifted provinces, such as West Antarctica, an additional correction accounts for the subsidence caused by mantle cooling (*T*_m_) or convection^78^. Using the age of rifting, the time of post-rift sedimentation, and a crustal stretching factor, thermal subsidence (*T*_S_) is determined. Tectonic structures active during the burial history can be considered when restoring the paleo-depths. In glacial environments, a final correction for glacio-isostasy is applied to the backstripping maps to account for both the eustatic and regional sea-level changes that result from the growth or decay of an ice sheet. Finally, cross comparisons between restored paleo-depths, seismic facies (e.g., grounding zone wedges) and lithofacies from drill sites are crucial to validate the backstripping applied to the sedimentary units. In fact, the interpretation relies on a number of parameters and physical simplifications that approximate water, sediments, lithosphere, and mantle rheological properties and behavior^52^.

## Interactions between bathymetry and open-ocean circulation

**Fig. 4:**
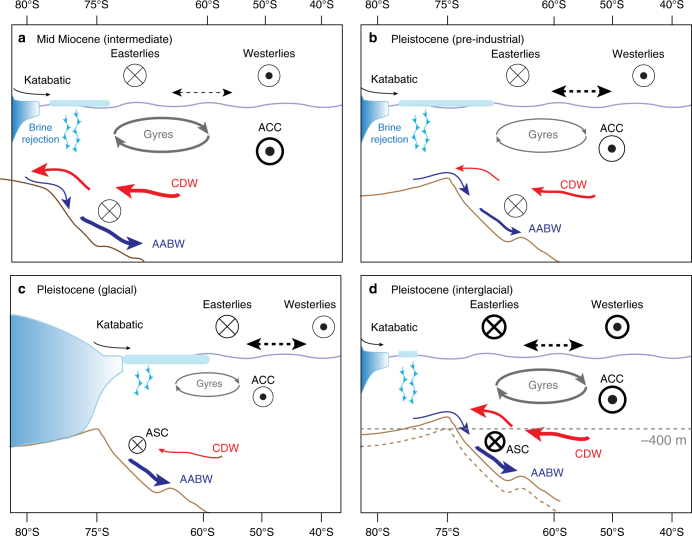
Schematics of bathymetric control on open-ocean circulation. Impact of long-term continental shelf expansion on the Southern Ocean high-latitude circulation in the Weddell Sea during the mid-Miocene **a**, during the late-Pleistocene **b**, and under modern-like climate conditions. During mid-Miocene (**a**), high-latitude ocean circulation is shifted southward due to the smaller continental shelf break^63^ compared with the modern one (**b**). Westerly winds are almost aligned with ACC, which strengthens the Gyre circulation and amplifies the inflow of CDW and outflow of AABW^89^ across the shelf break, also favored by stronger Easterly winds. At glacial/interglacial timescale, the incursions of CDW on the continental shelf are controlled by the depth and morphology of the continental slope and break^97,117^. During glacial periods **c**, with low atmospheric CO_2_ (< 200 p.p.m.), the AIS expands and the Westerly winds shift northward. Ice-sheet advance on the continental shelf inhibits oceanic circulation, which limits the incursions of CDW^118^. During ice-sheet retreat from the continental shelf edge, shallow-shelf ocean circulation is restored. Model simulations of super interglacials suggest that Westerly and Easterly winds are strengthened and are shifted polewards compared to their modern position **d**. This enhances incursions of CDW on the continental shelf, increasing the heat supply to the AIS grounding line, and promoting the formation and export of relatively fresh AABW^94^. However, depending on the depth of the continental shelf break and the strength of the Easterly winds modulated by atmospheric teleconnections, CDW may or may not intrude on the continental shelf, despite warm conditions

The role of ocean heat supply to AIS margins is a key aspect of ice-sheet vulnerability to global warming. Nowadays, oceanic measurements show how subsurface water masses enter into floating ice-shelf cavities inducing melting from below^9,79^. Limited geological evidence for such processes exists, documenting grounded ice sheet retreat as ocean temperatures have risen^80^. However, the mechanisms, spatio-temporal scales, and magnitude of ocean heat and salt transport onto and across the shallow Antarctic continental margins, and into marine embayments, remain poorly understood. Several factors complicate the interaction of warm ocean waters and ice shelves as follows: (1) the geometry and draft of the ice-shelf base; (2) water properties; (3) circulation in the sub-shelf cavity (to be discussed in upcoming reviews by Smith et al. in preparation); and (4) the connection to water masses outside of the cavity (Fig. 1 and focus of the present section). As in the case of subglacial hydrology and ice-sheet dynamics, bed morphology exerts a fundamental control upon each of these factors^81^ and must have done so in the geological past. In particular, bathymetry modulates Southern Ocean heat transport to the Antarctic continental slopes and shelves over the following three main spatial and temporal scales (Fig. 4).

### Large-scale ocean pathway geometries

The Antarctic Circumpolar Current (ACC) position is, to a great extent, constrained by the bathymetry of the ocean basins and gateways, and by the topography of the continental landmasses that affects the position of the local maximum of the Westerly winds^82^. The long-term bathymetric evolution of the basins that accommodate circumpolar circulation is adequately known and reveals that the gross circumpolar circulation systems within the Southern Ocean basins were established in the Eocene/Oligocene Epoch (≈ 40–25 Ma) with the opening of the Tasmanian Gateway and the deepening of Drake Passage^83^. Climate simulations of the EOT have shown that the opening of the gateways did not substantially change the moisture supply to the AIS enough to explain its complete glaciation^84^, but the gateway opening did contribute to a large-scale cooling, leading to the gradual expansion of ice sheets^85^.

Although the mean position of the ACC is controlled by long-term changes in ocean gateways, its strength and vigor depend upon the strength and relative position of the Westerly winds, in turn determined by the mean climate state. In general, a warmer climate is associated with a southward shift and strengthening of the Westerly winds^86^, leading to a more vigorous ACC, enhanced advection and volume of Circumpolar Deep Water (CDW), and strong-bottom Ekman transport and vice versa under cold climate conditions^82^. An intensification and poleward shift of near-surface ocean winds, attributed to positive Southern Annular Mode-like trends (atmospheric teleconnection) is projected for warmer climates by most climate models by 2100 ^86^. However, Coupled Model Intercomparison Project Phase 5^87^ (CMIP) and Paleo-modeling Intercomparison Project phase 3^88^ (PMIP) reveal a large spread in simulated strength of the ACC among models, suggesting that the position of the Westerly winds in climate simulations is model-dependent. This is of consequence, because heat transport to southern latitudes is in part regulated by the strength of the ACC.

### Position of continental shelf break

**Fig. 5:**
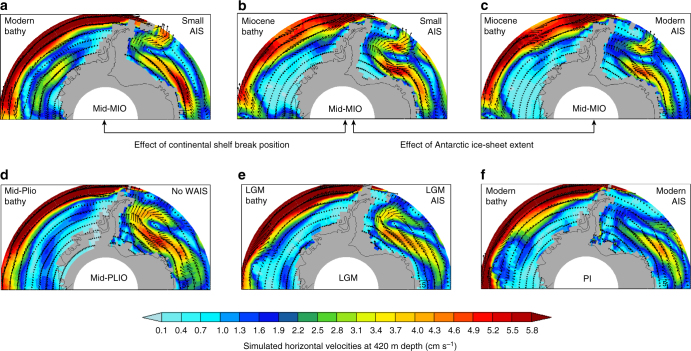
Impact of extent and location of the continental shelf break on ocean circulation for different mean climate states. Simulated ocean horizontal velocities (cm s^−1^) at a depth of 420 m (averaged core depth of CDW) for: **a** mid Miocene (mid-MIO)^89^, for modern continental shelf break location, small AIS extent and atmospheric CO_2_ concentration of 450 ppm representing 17–15 Ma^73^. **b** Same as for **a**, but with a smaller continental shelf break^63^ than in **a**. **c** Same as for **b**, but with modern AIS extent and atmospheric CO_2_ concentration of 278 p.p.m. representing 14–12 Ma. Comparison between **a** and **b** gives the impact of the continental shelf extent and location, whereas comparison between **a** and **c** shows the impact of AIS extent (and CO_2_ concentration, which is of minor importance). Bottom row: simulated mean ocean states for key periods of the Plio/Pleistocene. **d** Mid-Pliocene simulation (mid-PLIO, ≈ 3 Ma) with Pliocene bathymetry, an almost ice-free West AIS and atmospheric CO_2_ concentration of 405 p.p.m.^119^. **e** LGM ( ≈ 21 ka) with LGM  bathymetry, LGM AIS extent and atmospheric CO_2_ concentration of 185 p.p.m.^120^. **f** Pre-industrial simulation (PI) with modern bathymetry and AIS extent, and with atmospheric CO_2_ concentration of 278 p.p.m.^119^. The final 100 years of simulation of each model experiment is taken for analysis and outputs are available for download on the PANGEA database at https://doi.pangaea.de/10.1594/PANGAEA.889138

In models, the location of the continental shelf break determines the position of regional oceanic circulation systems such as the Weddell Sea and Ross Sea gyres (Fig. 4a, b). The models reveal how ocean flow under different climate conditions (present, glacial, Pliocene, and Miocene) and continental shelf extents is affected by topographic steering (Fig. 4). The ocean flow controls the amount of heat and salt, as well as nutrients, transferred from open ocean to the continental shelves^89^. Coupled atmosphere–ocean model simulations produce a more vigorous Weddell Sea gyre, with a southward shift from its current location, for times in the mid-Miocene (≈ 14–17 Ma) when the continental shelf break was located more southerly (Fig. 5a) than today (Fig. 5b). When this happens, heat transport onto and out from the Antarctic continental shelf is enhanced, with stronger Antarctic Bottom Water (AABW) formation. Independent paleoceanographic data suggest that AABW was the dominant source of global deep water until about 12 Ma^90^. Then, as the continental margin prograded northward close to its modern position, the model simulations show that AABW formation weakened. In late Miocene simulations, when a modern ice sheet is imposed, the simulated strength of the AABW inflow in the Atlantic Ocean is strongly modulated by the ice-sheet height that affect the winds and hydrography^89,91^. In such cases, the location of the continental shelf break is of minor importance for the ocean circulation (Fig. 5c).

Changes in ocean circulation on shorter timescales thereafter resulted from orbital effects, atmospheric CO_2_ forcing and sea-ice cover change. The degree of local bathymetric control upon ocean circulation is strongly dependent on the mean climate state. Furthermore, during the last deglaciation and the Pliocene warm periods, paleoceanographic data point to increased upwelling of CDW and incursion onto the continental shelf on suborbital timescales^80,92,93^ (Fig. 4d). If sea-ice cover is absent or reduced over the Antarctic continental shelves, freshening of the sea surface, along with strong Easterlies, facilitates upwelling of relatively warm CDW by increasing southward Ekman transport^94^, consistent with simulated mid-Pliocene ocean dynamics (Fig. 5d). During glacials, the subsurface ocean circulation cannot reach the continental shelf break (Fig. 5e) because of the topographic steering of the flow, and also because of the ice-sheet expansion that reaches almost the shelf edge (Fig. 4c). Due to strong winds and enhanced sea-ice formation during glacials relative to pre-industrial conditions (Fig. 5f), a pronounced formation of relatively cold and salty AABW is detected^95^.

### Morphology and curvature of continental slope

The depth of the continental shelf break and its position with respect to wind systems, as well as the steepness and curvature of the slope, lead to enhanced vertical dynamics and consequent exchange between warm CDW and newly formed AABW^96^. At a local scale, the heat and salt exchange across the shelf break does not occur continuously in time and space. On the one hand, incursions of CDW are controlled by the depth and the curvature of the continental shelf break^97^, as well as by the concavity or convexity of the isobaths along the slope, as, e.g., with the presence of incised sub-marine canyons^98^, which locally favor upwelling of warm water when cold water sinks downslope. On the other hand, the intensity and position of the Easterlies enhance the strength of the ASC geostrophic current, which might inhibit the upwelling and incursions of CDW across the continental shelf break and the outflow of AABW downslope^96^. Episodically, when Easterly winds weaken and bottom water accumulates on the continental shelf, overflow occurs and allows for the inflow of CDW. The main circulation path is also modulated at regional scale by mesoscale eddy activity. Those slope processes are essential to the AABW formation; however, according to model grid resolution, the steepness and morphology of the continental slope can be misrepresented. As a consequence, models might not capture adequately the overflow of AABW from the shelf across the shelf break and downslope, and the subsequent strongly baroclinic inflow of CDW.

High-resolution bathymetry and accurate paleobathymetric reconstructions are clearly needed to properly capture kilometer-scale oceanic processes leading to heat and salt exchange at the shelf break^99^. The lack of resolved bathymetry in models creates an incorrect heat transport across the continental shelf and incorrect sub-shelf melting, which in turn might induce an incorrect grounding line responses. However, this is a challenge, because high-resolution simulations cannot be integrated over the millennial timescales that are needed to account for long-term heat transport at global and regional scale. In the absence of pan-Antarctic high-resolution coupled ice-sheet-ocean models, we must learn about the AIS response to ocean warming from stand-alone circum-Antarctic^99^ or regional ocean implementations (such as the Weddell Sea^100^ or the Ross Sea^101^), or from physically based sub-shelf melting parameterizations in stand-alone ice-sheet models^102^. Those implementations are nevertheless useful to investigate how, under warmer than present mean climate states, ice sheets display threshold behavior in landward-deepening subglacial basins in response to relatively short-lived high-intensity ocean heat supply^103,104^.

## A unified model of Antarctic processes from past to future

**Fig. 6:**
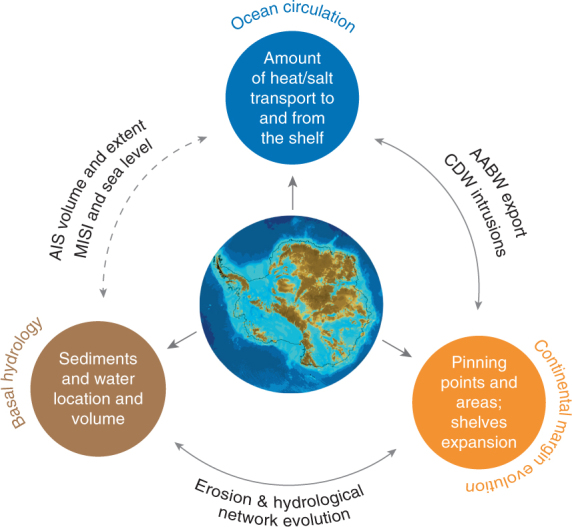
Main gaps identified for the three realms discussed in the review (colored circles). Each major gap spans present-day and past processes, and results from the lack of information about past and present topography and bathymetry (at the center), which severely limits our understanding of future AIS evolution. The largest unknowns are the processes and interactions at the interfaces between each realm (arrows), which urgently require cross-disciplinary research. The impact of sub-shelf ocean circulation on ice-sheet dynamics across the grounding zone (dashed arrow) is one of the least understood feedbacks and could contribute to processes leading to MISI

This review provides insights into processes operating at the interfaces between the ice sheet, its bed, the ocean and the continental margins around Antarctica (Fig. 1), all of which have been identified as central to research questions posed by the Scientific Committee on Antarctic Research (SCAR) Horizon Scan^105^. It highlights the processes least understood, poorly investigated or not implemented in models, that are understood to operate in a connected manner (Fig. 6). Atmospheric and solid Earth processes may also come into play. For example, an interplay between long-term faulting and shorter term differential erosion^49,106^ may influence the ice flow and lead to formation of pinning areas that have a stabilizing effect on the ice shelves, and therefore on the ice sheet, during both advance and retreat across the continental shelf. The regional and local morphology of the shelf break (interplay between curvature of the slope, as well as concavity and convexity of the isobaths) regulates the long-term and short-term heat/salt (AABW outflow and CDW inflow) and carbon/nutrients transfer across the shelf break and the ice-shelf cavities. The review also draws attention to the short-term interactions between ocean circulation in the ice-shelf cavities and ice-sheet dynamics across the grounding zone, which are seldom observed and largely unknown in Antarctica. These interactions potentially induce MISI. Although those processes may not operate on policy-relevant timescales, the ability to predict the AIS response to ocean warming and freshening, including the surpassing of tipping point(s) and contribution to global mean sea level, relies on advances in our understanding of interrelated processes and the feedbacks spanning the three realms in the past, present and future.

Due to the lack of integrated observations of the AIS basal processes and ocean circulation, there is little direct knowledge of the precise range of temporal and spatial scales of interactions between these realms. The knowledge gap has consequences for climate and ice-sheet model development, and experimental design strategies, because observations are needed to validate those recently developed or to develop new parameterizations of ocean–ice-sheet interactions^107^. High spatial and temporal resolution bed data are needed for models that simulate ocean circulation, including mesoscale eddy transport, basal hydrology, and marine-based ice-sheet dynamics, at the present-day margins of the ice sheet. Such data are also required to identify regions of Antarctica where the grounding line may migrate under atmospheric and ocean warming.

To quantify AIS contribution to past, present and future global mean sea-level variations, paleo-polar-change and contemporary polar-change communities must converge toward the same objective, which is to identify precursor signals to MISI or AIS tipping points, with substantiated timescales. Investigating the AIS sensitivity to climate changes, whether it is in the past, ongoing, or projected, requires the study of the grounding line response to a variety of external perturbations, such as sea-level rise, atmospheric and oceanic warming, local to regional wind effects (katabatic winds and polynyas), sub-shelf circulation changes and subglacial hydrology influences (Fig. 1). Timescales of grounding line response to ice-sheet advance, MISI, or ice-sheet retreat may span several thousands of years to a few years or centuries. Conversely, ice-sheet response to atmospheric or oceanic warming and circulation changes can span centuries or millennia (e.g., shift in position or strength of atmospheric cells, amount of deep water formation) down to seasonal/decadal timescales (e.g., teleconnections, sea-ice extent and volume, and sea surface temperature inter-hemispheric meridional and zonal gradients). The spatial framework for MISI and for grounding line advance or retreat is regional to local, whereas atmospheric or ocean circulation and heat transport changes are affected by processes that act both at the local scale and regional to global scales.

**Fig. 7:**
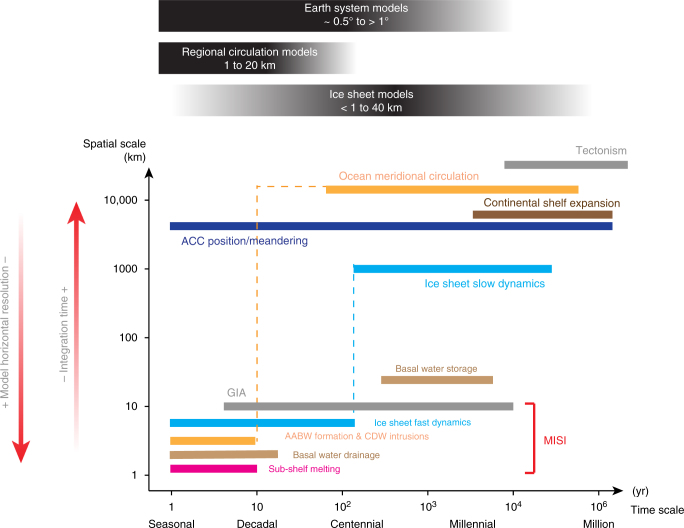
Spatio-temporal scales of the processes discussed in the present review. Short-term processes occurring at local to regional scales are the ones potentially triggering MISI. Heat transport from open ocean to the continental shelf also depends on the long-term meridional overturning circulation (dashed orange line). Similarly, short-term ice sheet dynamics, and their response to local forcing, depend partly on its long-term evolution. The difficulty for the modeling community resides in capturing the long-term essence of those processes, occurring at continental to global scale and short-term response occurring at local to regional scales within the same simulation. A trade-off between numerical model horizontal resolution and integration time is so far still necessary. Note that vertical resolution of numerical models is not mentioned but is essential to capture the continental slope and shelf oceanic processes (heat, salt and nutrient transport). No atmospheric processes are reported here but they are implicitly contained in the AABW formation, CDW intrusions, ACC position and meandering (winds effect, polynya opening, etc.) and in ice-sheet short and fast dynamics (surface mass balance processes) as described in the previous sections. Note that glacio-isostatic adjustment (GIA) and sub-shelf melting are to be discussed in upcoming reviews by Smith et al. (in preparation) and Whitehouse et al. (in preparation). Tectonism refers to volcanism and crustal deformation of timescales that are beyond the ones discussed in this review

One of the major challenges for the polar community to surmount is the representation of the interplay of long-term, large-scale processes, and small-scale, short-term processes from both observational and modeling points of view (Fig. 7). Processes occurring within the ice-shelf cavities are emblematic of this interplay, insofar as they span short timescales of hours (e.g., tides) to decades (e.g., inflow of ocean warm water or seasonal water masses) on local scales of a few kilometers (e.g., grounding lines). Knowledge of the cavity environment is imprecise because of the scarce spatial and temporal data coverage for the present-day circum-Antarctic ocean and sub-shelf circulation, and for geological proxies that inform about past cavity conditions. However, processes within the cavity may impact on the overall AIS dynamics and may have long-term consequences on AIS volume, potentially inducing large-scale changes in the global climate system and vice versa (Fig. 7). Therefor, it is paramount to acquire geological proxies for paleo-oceanic conditions at sufficient temporal and spatial resolution for direct comparison with present-day observations^108^.

High-resolution regional simulations, and long-term simulations of the past, entail significant computational cost, so the capability of supercomputing centers must increase and/or model codes must be optimized for computation of essential feedbacks. The use of unstructured grids in climate and ice-sheet models is a good example of optimization and is under development. It allows for nested areas of horizontal mesh refinement to or higher than 1 km (at least on a regional scale)^100,109,110^, which will allow coupled regional or global models to optimize computational resources, while simulating processes and feedbacks that occur at a variety of spatial and temporal scales.

To support high-resolution modeling, improved continental shelf and sub-shelf bathymetry (among other quantities) is a priority. Updated and new near-shore R-TOPO2 bathymetry for the circum Antarctica^111^, the ongoing ROSETTA-Ice Project (Tinto et al. (in preparation); http://www.ldeo.columbia.edu/res/pi/rosetta/), whose objective is to retrieve the bathymetry beneath the Ross Ice Shelf, and forthcoming BEDMAP3, reflect continued progress in gradually filling this gap. High-resolution modeling that investigates past AIS-ocean interactions during key intervals, such as the mid-Miocene (≈ 14 Ma), mid-Pliocene (≈ 3 Ma), and some of the late Pleistocene interglacials, is also needed. The reconstruction of realistic past subglacial bed and seabed morphologies, which persists as a major challenge, is one of the major objectives for the SCAR scientific research program Past Antarctic Ice Sheet dynamics (PAIS) (https://www.scar.org/science/pais/).

Strong research synergies have developed between the paleo and present-day observational and modeling communities over the last two decades. Major community initiatives such as CMIP, now in phase 6 (present day and future), which includes for the first time the Ice Sheet Model Intercomparison Project 6 (sea-level contribution from the Greenland and Antarctic ice sheets^112^), the PMIP now in phase 4^113^, the PLIOcene Model Intercomparison Project^114^ (LGM, last interglacial, mid-Holocene, and mid-Pliocene), and the Pliocene Ice Sheet Modeling Intercomparison Project^17^, have highlighted the large range of climate and ice-sheet projections that result from gaps in the model physics and observations. In developing a regional polar system model for the past, present, and future, improvements in global climate models are required. This can only be achieved if present-day and paleo-climate data coverage improves, both in the polar areas and in the representation of the essential teleconnections between the equatorial region and Antarctica. Our understanding of the Polar system requires strong synergies among the polar and global climate communities to investigate interrelated processes and feedbacks in a common framework aimed at assessing the onset and surpassing of tipping point(s), and global mean sea-level changes in the past, present and future.
