# Publisher Correction: Spatio-temporal variability of processes across Antarctic ice-bed–ocean interfaces

**DOI:** 10.1038/s41467-018-05206-4

**Published:** 2018-07-11

**Authors:** Florence Colleoni, Laura De Santis, Christine S. Siddoway, Andrea Bergamasco, Nicholas R. Golledge, Gerrit Lohmann, Sandra Passchier, Martin J. Siegert

**Affiliations:** 1 Fondazione Centro Euro-Mediterraneo sui Cambiamenti Climatici, 40129 Bologna, Italy; 2 Istituto Nazionale di Oceanografia Sperimentale, 34010 Sgonico, Italy; 3 Department of Geology, Colorado College, Colorado Springs, CO 80903 USA; 4 Centro Nazionale delle Ricerche - Istituto di Scienze Marine, 30122 Venice, Italy; 5 Antarctic Research Centre, Victoria University of Wellington, Wellington, 6140 New Zealand; 6 GNS Science, Avalon, Lower Hutt, 5010 New Zealand; 7 Alfred Wegener Institute, Helmholtz Centre for Polar and Marine Research, 27570 Bremerhaven, Germany; 8 University of Bremen, 28359 Bremen, Germany; 9 Department of Earth and Environmental Studies, Center for Environmental and Life Sciences, Montclair State University, Montclair, NY 07043 USA; 10 Grantham Institute and Department of Earth Science and Engineering, Imperial College of London, London, SW7 2AZ UK

Correction to: *Nature Communications*; 10.1038/s41467-018-04583-0; published online 18 June 2018

The original version of this Article contained an error in the spelling of the author Florence Colleoni, which was incorrectly given as Florence Colloni. This has been corrected in both the PDF and HTML versions of the Article.

